# A web-based approach to adolescent mental health: Randomized controlled trial of a brief Positive Psychology intervention

**DOI:** 10.1016/j.invent.2025.100872

**Published:** 2025-09-12

**Authors:** Sara Kaubisch, Maria Kloek, Regine Primbs, Lucia Iglhaut, Charlotte E. Piechaczek, Pia-Marie Keim, Lisa Feldmann, Gerd Schulte-Körne, Ellen Greimel

**Affiliations:** a Department of Child and Adolescent Psychiatry, Psychosomatics and Psychotherapy, LMU University Hospital, LMU Munich, Munich, Germany; b German Center for Mental Health (DZPG), Partner Site Munich-Augsburg, Munich, Germany

**Keywords:** positive psychology, prevention, mental health, well-being, web-based, adolescents, intervention

## Abstract

**Theoretical background:**

The high prevalence of mental health problems as well as the substantial rise in the prevalence of major depression among young people is a major concern worldwide. There is an urgent need for easily accessible interventions that promote well-being and mitigate mental health problems in adolescents before mental health problems worsen. Hence, we developed a freely accessible, brief online intervention based on Positive Psychology for youth.

**Objective:**

This randomized controlled trial (preregistered at ClinicalTrials.gov: NCT04994496) examined the efficacy, acceptance, and adherence of a brief online Positive Psychology intervention to improve affect- and stress-related outcomes in healthy adolescents in comparison to an active control condition.

**Methods:**

79 adolescents aged 12 to 18 (*M* = 15.65, *SD* = 1.74) were randomly assigned to the experimental group, which received 14 daily web-based self-help exercises based on Positive Psychology, or to the control group, which received a web-based active control intervention (factual messages unrelated to Positive Psychology). Changes in affect- and stress-related outcome measures as well as acceptance of the intervention were assessed using self-report inventories. Adherence to the intervention was measured using objective indicators and self-reporting.

**Results:**

There were no differential effects of the Positive Psychology intervention on affect- and stress-related outcomes compared to the control group. The overall acceptance of the Positive Psychology intervention was good and more than 83 % of the participants in the Positive Psychology intervention group reported that they would recommend the exercises to other adolescents. Furthermore, more than 87 % of the adolescents in the Positive Psychology intervention group reported that they carried out the exercises, and usage data showed that approximately 64 % opened 10 or more of the links that contained the exercises.

**Conclusion:**

The findings have important implications for future efforts in the prevention of mental health problems. In particular, they provide more information on how to deliver brief online, multi-component Positive Psychology interventions for healthy young people. As the results indicated good acceptance and adherence in our adolescent sample but no differential effects, we encourage further mixed methods research evaluating the perceived usefulness and person-activity-fit to understand the optimal methodology for the delivery of Positive Psychology interventions to have beneficial effects.

## Highlights

• Randomized controlled trial on the efficacy, acceptance, and adherence of a Positive Psychology intervention in adolescents
• No differential effects on affect- and stress-related outcome measures were revealed
• The intervention showed high acceptance among adolescents
• A good level of adherence was demonstrated based on both objective and subjective indices
• Results emphasize the need for further research into the mechanisms of action

## 1 Introduction

The high prevalence of mental health problems among young people is a major global concern, with 11.63 % of children and adolescents worldwide suffering from a mental disorder (Kieling et al., 2024). This includes depressive disorders, which belong to the most common mental disorders in children and adolescents worldwide (Sacco et al., 2024), with prevalence rates of up to 8 % in adolescence (Shorey et al., 2022). Over the past decade, there has been considerable concern about the substantial rise in the prevalence of major depression (MD) among adolescents (Steffen et al., 2020; Thapar et al., 2022). More recently, in the context of the COVID‐19 pandemic, a meta-analysis found an increase in clinically relevant depressive symptoms among youth to over 25 % (Racine et al., 2021).

Given the concerning prevalence of mental health issues as well as the increasing prevalence of depressive symptoms and disorders in youth, there is an urgent need for approaches that focus on psychoeducation and support to mitigate mental health problems, including depressive symptoms, in adolescents before they deteriorate (Shorey et al., 2022). Mental health is increasingly understood as a state that involves both the presence of well-being and the absence of mental illness (Campion et al., 2012; Westerhof and Keyes, 2010). It is emphasized that the absence of mental illness does not necessarily indicate the presence of positive mental health (Campion et al., 2012; Keyes, 2005). This distinction highlights the importance of promoting positive mental health alongside preventing mental disorders (Campion et al., 2012; Tejada-Gallardo et al., 2020). Traditional interventions often focus on reducing mental illness, but promoting resilience and well-being is equally important for achieving long-term health and social benefits (Campion et al., 2012; Lyubomirsky et al., 2005). One promising avenue comprise low-barrier, location-independent, highly scalable interventions and access to resources that help promote well-being and reduce psychopathology.

A resource that covers the promotion of well-being and the prevention of mental disorders like MD is Positive Psychology interventions (PPI). Positive Psychology (PP) focuses on the research into which personal strengths, abilities, and resources can be targeted to foster well-being (Seligman et al., 2006). In terms of intervention mechanisms, PPIs were traditionally defined as “treatment methods or intentional activities that aim to cultivate positive feelings, behaviors, or cognitions” (Sin and Lyubomirsky, 2009). There are currently numerous PPIs available - with established components such as gratitude, strengths, or acts of kindness (e.g., Lyubomirsky et al., 2011; Seligman et al., 2005). The “Positive Pathways to Health Theoretical Model” (Moskowitz et al., 2019; Moskowitz et al., 2021) suggests that PPIs increase the frequency of positive emotions, which is claimed to have several beneficial effects, such as the promotion of more adaptive coping strategies, the reduction of emotional reactivity to daily stress, and the strengthening of relationships. This, in turn, is thought to lead to a reduction in stress, along with an improvement in physiological functioning and a greater commitment to preventative health behaviors. According to the model, this ultimately improves psychological (and physical) well-being, including, for example, less depression and anxiety, as well as greater life satisfaction.

In their systematic review, Donaldson et al. (2021) summarized the results from high-quality randomized controlled trials (RCTs) in which the efficacy of PPIs in studies involving mostly adult populations, both clinical and non-clinical, and some involving clinical and non-clinical adolescents were investigated. The quality of these studies had already been assessed in previous reviews and meta-analyses. The authors conclude that PPIs are effective, with effect sizes varying between small and medium for outcomes such as well-being, depression, anxiety, stress, and quality of life for the general population and patients suffering from mental disorders. However, taking into account the bias caused by small sample sizes, the effect sizes of PPIs on well-being are only small, but there is still a significant improvement of well-being (approximately *r* = .10, White et al., 2019, predominantly adult samples).

While the majority of studies have concentrated on the effects of single-component PPIs (Lambert et al., 2019), it has been proposed that providing various activities rather than a single one can enhance the overall efficacy (Schueller and Parks, 2012; Hendriks et al., 2020). Multi-component PPIs target two or more theoretically related well-being components (such as gratitude and strengths), rather than focusing on just one component. In their systematic review and meta-analysis of psychological interventions to improve mental wellbeing in healthy, mentally, and physically ill populations, van Agteren et al. (2021) found that specifically multi-component PPIs (next to mindfulness-based or cognitive and behavioural therapy-based interventions, CBT) belong to the psychological interventions that show the greatest efficacy with small to moderate effects on improving well-being.

Studies on the efficacy of PPIs and multi-component PPIs in adolescents are still scarce, albeit their number has been increasing in recent years. The majority of studies that researched the effects of PPIs in adolescents focused on the school setting and provided evidence that PPIs decrease depression and anxiety symptoms as well as increase positive emotions and well-being as shown in two meta-analyses (Owens and Waters, 2020; Tejada-Gallardo et al., 2020).

Youth as so-called “digital natives” strongly prefer to be self-reliant in handling their mental health (Gulliver et al., 2010) and they use the internet to seek mental health information (Scott et al., 2022). Therefore, digital technologies are increasingly being utilized to address the mental health promotion needs of this age group (Bergin et al., 2020) and a growing number of research indicates their potential (Wright et al., 2023). Web-based or online PPIs have been shown to promote well-being and reduce depressive symptoms in healthy adults and those with depressive symptoms (see Mitchell et al., 2010, for a review). A narrative review of online PPIs in young people (Baños et al., 2017) identified 9 RCTs including multi-component and single-component PPIs and found positive effects of online PPIs in the form of an increase in well-being and a decrease in depression and anxiety scores. However, as almost no longitudinal studies could be identified, the authors point out that more controlled studies with (long-term) follow-up are needed. Moreover, the review highlights that PPIs should be designed with consideration of the needs of the target group and the context in which the PPI will be implemented (like school, family, leisure, etc.). This is crucial to ensure the engagement of adolescents. In addition, given that investigating the acceptance of digital interventions is vital for overcoming concerns or barriers that users may encounter (Yardley et al., 2015), some of the studies researching PPI have addressed this aspect and found a high level of acceptance (e.g., Manicavasagar et al., 2014).

## 2 Current study

Research to date collectively provides evidence for the efficacy of PPIs in increasing well-being and reducing distress in clinical and non-clinical adult and adolescent samples (see for a review, Donaldson et al., 2021). However, there is currently little research on the efficacy and acceptance of multi-component PPIs in adolescents, especially in an online setting. Thus, well-controlled studies among adolescents with a sufficient sample size are needed to determine whether online multi-component PPIs are effective and accepted by the target group. Therefore, the current study sought to address this gap by investigating the efficacy, acceptance, and adherence of a brief online multi-component PPI among adolescents on well-being, including affect measures (Hendriks et al., 2020) as well as depressive symptoms and stress as indicators of mental health (Bolier et al., 2013) against an active control condition. We defined efficacy as primary outcome and acceptance and adherence as secondary outcomes. Based on previous research with predominantly non-clinical adult (Bolier et al., 2013; Sin and Lyubomirsky, 2009; Hendriks et al., 2020), clinical adult (Chakhssi et al., 2018), and adolescent samples (Tejada-Gallardo et al., 2020), it was hypothesized that, compared to the active control group, a two-week online multi-component PPI would (1) increase positive affect, (2) lower negative affect, (3) reduce stress, and (4) alleviate depressive symptoms in healthy adolescents. We also investigated the rates of adherence among the adolescents who used this web-based intervention and the acceptance of the intervention. As the online multi-component PPI was developed based on a participatory approach (including, e.g., interviews with adolescents about design aspects and the tonality of the content), we expected a good adherence and acceptance.

## 3 Methods and materials

### 3.1 Study design and setting

The current study was conducted as an RCT over two weeks with a two-week follow up. To systematically evaluate the online PPI in the current study a parallel group, participant-blinded design was employed, in which 14 PP exercises were compared with an active control intervention consisting of 14 interesting facts. Both interventions were provided via a daily email over a two-week period. The PPI evaluated in this study forms part of the German information platform on mental health and depression in youth “ich bin alles” (English: “I am everything”; www.ich-bin-alles.de). More information on the website can be found in the Supplementary Material. In the context of “ich bin alles”, selected contents of the website were scientifically evaluated in healthy adolescents, adolescents with MD, and the parents of these groups (Iglhaut et al., 2024; further studies are referenced under Clinical Trials.gov NCT05300204, NCT05300217, NCT05326178). Building on a previous study in adolescents with MD (Kaubisch et al., 2023), this paper outlines the study aiming to systematically evaluate the PP exercises in healthy adolescents in an experimental setting (preregistered at ClinicalTrials.gov: NCT04994496). In the current study, investigators (R.P. and L.I.), who were not involved in data collection, randomly assigned the participants to either the experimental group (EG), which received the PPI, or the active control group (CG), which received factual knowledge, using a 1:1 randomization stratified by sex and age (<15 years vs. ≥ 15 years). The investigators, who were assigned to collect data (including S.K.), were blind to the group assignment during the diagnostics, but not during the subsequent procedures, as they were responsible for sending the email with the interventions and assessing the group-specific questionnaires. Participants were kept blind until the end of data collection, where they were then informed about the different groups and objectives of the study. Data was collected between January 2021 to May 2022. The relatively long timeframe was due to the intensive diagnostic procedure (including structured clinical interviews) required prior to enrollment and restrictions related to the pandemic, which made it necessary to temporarily suspend in-person data collection due to national and institutional regulations. The study adhered to the current Declaration of Helsinki and national legislation, and received institutional review board approval. Written informed consent/assent was obtained from participants and their parents/legal guardians (for participants <18 years).

### 3.2 Participants

Adolescent participants were recruited through the Department of Child and Adolescent Psychiatry, Psychosomatics and Psychotherapy, LMU University Hospital, LMU Munich, via flyers, from a list of interested families who could be contacted for studies within the department, and via address lists obtained from the local registration offices. Participants were rewarded in the form of a voucher. Regarding inclusion criteria, participants were required to be 12 to 18 years old and have an intelligence quotient (IQ) of 80 or above, as assessed with the German version of the Culture Fair Intelligence Test (CFT-20-R; Weiß, 2006) to ensure comprehension of the presented content and questionnaires. Exclusion criteria included insufficient German language skills and a current diagnosis of a mental disorder and/or remitted depressive disorder based on ICD-10 and DSM-5. Trained staff members applied the Diagnostic Interview for Mental Disorders for Children and Adolescents (Kinder-DIPS; Margraf et al., 2017; Schneider et al., 2017), a standardized semi-structured German diagnostic interview, for diagnostic assessment of potential axis I mental disorders in youth according to ICD-10 and DSM-5. A diagnostic interview was chosen as part of the enrollment procedure to ensure the absence of a current mental health condition and a past depressive disorder which is important for a precise interpretation of the intervention's effect. Participants' current depressive symptoms were assessed using the Beck Depression Inventory - Second Edition (BDI-II; Hautzinger et al., 2006) and to assess stress, the German version of the Perceived Stress Scale (PSS-10; Schneider et al., 2020) was applied. However, we did not apply cut-off scores for BDI-II and PSS-10 as exclusion criteria. That is, adolescents with elevated scores on these questionnaires were included in the study as long as no diagnosis was established in the diagnostic interview. In cases of acute psychological distress (identified either through the clinical interview or via the suicidality item in the BDI-II) a standardized procedure was put in place that involved mental health professionals from the department's hospital to ensure appropriate follow-up and care. The level of education of the participants' parents (highest completed education, reflecting parents' school and professional qualifications) as an indicator of the socioeconomic status was measured based on Lampert et al. (2018), utilizing a parent-report questionnaire. After applying the exclusion and inclusion criteria and accounting for drop-outs, the final sample consisted of *N* = 74 healthy adolescents (see Table 1 for demographic characteristics). Based on an a priori power analysis, this sample size was deemed sufficient to test our hypotheses, as detailed in the Supplementary Material. Fig. 1 in the Supplementary Material presents the *Consolidated Standards of Reporting Trials* diagram outlining each evaluation stage.

**Table 1:** Descriptive statistics for demographic variables and baseline scores of the primary measures of the study sample (*N* = 74)

	Total (*N* = 74)	Experimental group: Positive Psychology intervention (*n* = 36)	Control group (*n* = 38)	Statistic	*p*
Sex (female), *n* (%)	40 (54.1 %)	19 (52.8 %)	21 (55.3 %)	0.05^a^	.830
Age, *M* (*SD*)	15.65 (1.74)	15.67 (1.61)	15.64 (1.87)	0.81^b^	.936
IQ, *M* (*SD*)	109.21 (13.42)	108.36 (13.98)	110.09 (12.97)^d^	0.57^b^	.592
Level of education, *n* (%)^c^				0.01^a^	.908
Higher	58 (77.3 %)	28 (77.8 %)	30 (78.9 %)		
Middle	14 (18.7 %)	7 (19.4 %)	7 (18.4 %)		
Lower	0 (0.0 %)	0 (0.0 %)	0 (0.0 %)		
PANAS-C-SF: Positive Affect, *M* (*SD*)	16.57 (3.51)	16.14 (3.10)	16.97 (3.85)	−1.02^b^	.309
PANAS-C-SF: Negative Affect, *M* (*SD*)	5.46 (1.38)	5.47 (1.46)	5.45 (1.31)	0.08^b^	.939
PSS-10, *M* (*SD*)	22.07 (6.09)	21.51 (6.93)	22.61 (5.21)	−0.77^b^	.445
BDI-II, *M* (*SD*)	4.05 (5.67)	3.83 (6.61)	4.26 (4.71)	−0.32^b^	.747

*Abbreviations:* PANAS-C-SF = Positive and Negative Affect Schedule for Children-Short Form; PSS-10 = Perceived Stress Scale; BDI-II = Beck Depression Inventory - Second Edition.

a Pearson-Chi-Square Test.

b Independent Samples *t*-Test.

c Level of parental education based on Lampert et al. (2018); *n* = 72 (because of missing data of 1 participant in each group).

d *n* = 35 (because of missing data).

### 3.3 Procedure and experimental materials

#### 3.3.1 Procedure

All assessments took place at the Department of Child and Adolescent Psychiatry, Psychosomatics and Psychotherapy, LMU University Hospital, at the Hospital of the LMU Munich. The initial appointment (T1; ∼2 h) started with detailed diagnostics including the Kinder-DIPS and CFT-20-R. After diagnostic assessment, participants completed baseline assessments (questionnaires assessing primary outcomes). After the first appointment, eligible participants were instructed to carry out the intervention every day for the following two weeks. The intervention was sent out through daily emails with PP exercises or emails containing text messages with the factual knowledge. The order of exercises/messages was predefined and the same for all participants in the experimental and control group, respectively (see Supplementary Material Tables 1 and 2). Participants received up to three reminder emails during the intervention period if a link (with the exercise/message) was not opened. The amount of sent reminders was comparable in the two groups (*M*_EG_ = 1.97, *SD*_EG_ = 1.25; *M*_CG_ = 2.08, *SD*_CG_ = 1.19; *t*(72) = −0.38, *p* = .709). After two weeks, post-intervention assessment (T2; ∼45 min) took place. At T2, affect- and stress-related primary outcomes were assessed again and acceptance as well as adherence as secondary outcomes were evaluated. After two more weeks, the follow-up appointment (T3; ∼45 min) took place, where the primary outcomes were reassessed. We allowed a goodwill period of up to 21 days between time points (T1–T2 or T2–T3) because of scheduling difficulties, especially due to the ongoing COVID-19 pandemic (e. g., due to quarantine), with two participants exceeding this period between T1-T2 and/or T2-T3. These participants were excluded from the analyses on primary outcome measures, but were included in acceptance and adherence analyses assessed at T2. Average intervals between T1 and T2 were 14.93 days (*SD* = 1.92; with no differences between groups, *t*(72) = −0.55, *p* = .583) and between T2 and T3 were 14.14 days (*SD* = 1.27; with no differences between groups, *t*(71) = 0.20, *p* = .846). Additionally, at T1, T2, and T3, participants answered whether they had visited the “ich bin alles” website (as the PPI is an additional part of the website; see Supplementary Material for more information). While three participants reported to have visited the website (1 from the EG at T2 and T3, 1 from the CG at T1 and 1 from the CG at T2), exploratory analyses showed that excluding these participants did not change result patterns for primary and secondary outcomes. Hence, results from the full sample are presented.

#### 3.3.2 Positive Psychology intervention

**Fig. 1:**
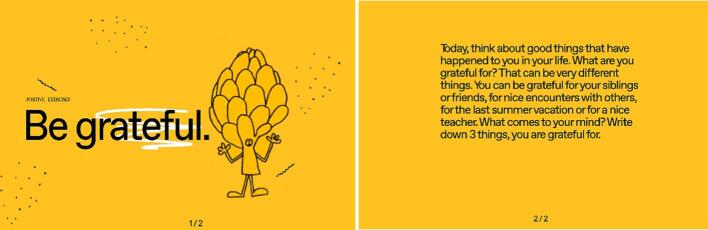
Translated example of a web-based exercise based on Positive Psychology (original is in German).

The brief web-based PPI includes 14 (partly) interactive exercises targeting various PP domains, such as gratitude, pleasure, personal strengths, positive relationships, and engagement including mindfulness and flow (detailed in Supplementary Material Table 1). These exercises were originally designed as an additional part of the “ich bin alles” website to engage youth by offering practical support in daily live, and to ultimately achieve greater acceptance of the website. The design of the exercises and the website was created to appeal to young people in particular, based on interviews carried out with youth prior to the website's conception. A colorful layout and a positive tonality were highlighted as relevant aspects. Participants could complete the exercises independently without additional support. To systematically evaluate potential beneficial effects of the PPI in this study, participants in the EG received daily emails for 14 days with links to the individual exercises. These links were created specifically for the study, so participants only performed one exercise per day. Thus, experimental control regarding intervention dose should be maximized (see Fig. 1 for an example exercise, originally in German, translated to English). In this e-mail, participating adolescents received a link to the daily self-help exercises (see Supplementary Material Table 1). At T1, they were instructed to complete one short exercise each day for 14 consecutive days, which would be delivered via email at 7 am. Participants were told that it was important to do the exercises. The emails did not provide any further information on how the interventions were to be carried out.

#### 3.3.3 Active control intervention

The web-based active control intervention consisted of 14 partly interactive text messages with factual knowledge content which were also sent out via email. These messages' contents were unrelated to PP and well-being (see details in Supplementary Table 2 and Supplementary Fig. 2). The layout and the lengths were chosen to be as similar as possible to the PPI and selected to be interesting and engaging for the target group. To ensure further comparability between the experimental and the control group the same delivery format was chosen for the exercises and the factual messages. The control intervention only differed from the PPI in content and interactivity: PP exercises focused on well-being-related topics (e.g., strengths, gratitude) and included brief interactive tasks (10 of 14), such as writing down reflections or speaking to a friend. In contrast, control messages contained non-related factual trivia (e.g., “Is a rainbow also visible at night?”) and offered multiple-choice (5 of 14) or open-ended quizzes with feedback and thus were less interactive. In the daily e-mails, the adolescents received a link to the daily factual message, but no further instructions on how to carry it out. Control participants received the same instructions as the adolescents in the PPI group at T1, except that they were told that they get messages rather than exercises.

#### 3.3.4 Primary outcome measures: assessment of affect, stress, and depressive symptoms

The primary outcome measures included positive and negative affect as indicators of well-being (Hendriks et al., 2020), and stress and depressive symptoms as mental health parameters (Bolier et al., 2013), assessed at three time points (pre-intervention, T1; post-intervention, T2; two-week follow-up, T3).

Positive emotion is thought to be one of the key mechanisms through which PPIs influence psychological health (Moskowitz et al., 2019; Moskowitz et al., 2021; Schueller et al., 2014). Therefore, the Positive and Negative Affect Schedule for Children-Short Form (PANAS-C-SF; Ebesutani et al., 2012) was applied to assess self-reported changes in momentary positive affect (PA) and negative affect (NA) on 5-point response scales, whereby higher scores indicate more PA or NA, respectively (range on both subscales 5–25). Subjective well-being is often indicated as a combination of high PA and low NA. The PANAS-C-SF was selected due to the frequent use of this instrument in research on PPIs with self-reported affective states (e.g., Celano et al., 2017; Coote and MacLeod, 2012).

Positive resources targeted by PPIs such as hope or gratitude can contribute to protect from stress and promote resilience (Ferrandez et al., 2022; Martínez-Martí and Ruch, 2017). To assess stress, the German version of the Perceived Stress Scale (PSS-10; Schneider et al., 2020) was applied. The PSS-10 is a self-report inventory with 5-point response scales to assess perceived everyday stress, with higher scores indicating more stress (range from 0 to 40).

The BDI-II (Hautzinger et al., 2006) was used to measure depressive symptoms, with this outcome measure being one of the main foci in the research on effectiveness of PPIs (Bolier et al., 2013; Sin and Lyubomirsky, 2009). Individual scores can range from 0 to 63. This instrument is normed for ages 13 and above but was applied to the entire sample for consistency as only four participants were aged 12 (for a similar approach see Feldmann et al., 2023, or Zsigo et al., 2024). Supplementary Material Table 3 provides detailed descriptions of the psychometric properties of the measures used.

#### 3.3.5 Secondary outcome measures: acceptance of and adherence to the intervention

Acceptance of the intervention was measured through a self-report inventory designed by the investigators. To assess overall acceptance, participants graded the exercises (“What grade do you give the positive exercises?”; grade 1: very good to grade 6: insufficient, based on the German school grading system). Table 2 summarizes the additional items on acceptance, to which participants responded on a four-point rating scale (0: not accurate to 3: entirely accurate).

**Table 2:** Results on acceptance and adherence for the Positive Psychology intervention (in %)

Items	Entirely accurate	Mainly accurate	Somewhat accurate	Not accurate
**Acceptance**				
I was able to understand the instructions for the positive exercises well.	**94.4**	5.6	0	0
It was fun to do the positive exercises.	**47.2**	44.4	8.3	0
I would recommend the positive exercises to other young people.	**41.7**	**41.7**	16.7	0
I was able to apply the positive exercises well in my everyday life.	36.1	**44.4**	19.4	0
The positive exercises have improved my mood.	16.7	**58.3**	25.0	0
The positive exercises have energised and activated me.	16.7	**47.2**	36.1	0
**Adherence**				
I performed the positive exercises.	37.5	**50.0**	8.3	4.2

*Note*: *n* = 36 (except for the item “I performed the positive exercises.” *n* = 24). Most frequent responses in bold.

Adherence to the intervention was assessed through usage data indicating how many email links participants opened. While opening a link does not confirm execution of exercises or reading factual messages, it is a prerequisite for these adherence aspects. Additionally, adherence was assessed based on two self-reported items. The PPI group rated the item “I performed the positive exercises.” on a four-point rating scale (0: not accurate to 3: entirely accurate). Participants also answered the question “How many positive exercises have you done?” with options “None.”, “Approx. 1−4 positive exercises.”, “Approx. 5−9 positive exercises.”, “Approx. 10−13 positive exercises.”, and “All.”. As the items on adherence were added shortly after the start of the study, some data (*n* = 12) are missing. Details for the items regarding acceptance and adherence for the control group are provided in Supplementary Table 4.

### 3.4 Data analysis

To check whether randomization was successful, we tested for differences between the baseline characteristics of the two groups in terms of sex distribution, age, IQ, level of education of the participants' parents, and baseline scores of the primary outcome measures based on independent-samples *t*-test and Chi-square tests. To analyse the effect of the interventions on primary outcomes, mixed ANOVAs with time (T1/T2/T3) as within-subject factor and group (EG/CG) as between-subject factor were conducted. Greenhouse-Geisser correction was applied if sphericity was violated (Mauchly's test). Partial eta square (η^2^) was computed for all ANOVAs. Significant main or interaction effects prompted post-hoc *t*-tests. For acceptance and adherence outcomes in both groups, descriptive statistics were calculated. With regard to the usage data pertaining to adherence, participants with lower link-opening rates were retained in the analyses to avoid bias (*Revised Cochrane risk-of-bias tool for randomized trials;* Higgins et al., 2019). However, 2 adolescents were excluded because they did not carry out the intervention (forgot the intervention and for unknown reasons). In this context, we exploratively analysed whether the extent of adherence as indicated by objective usage data influenced primary outcomes. We explored this dose-response by dividing the EG into complete (all 14 exercises) and incomplete usage groups, and conducted independent *t*-tests comparing these groups using the difference scores in the primary outcome measures between T1 and T2. These exploratory analyses revealed no significant differences in the primary outcomes (BDI-II, PSS-10, PANAS-PA, PANAS-NA) between the groups, i.e., no dose-response relationship was revealed based on the number of PP exercises completed.

12 EG and 19 CG participants continued to (re)open links between T2 and T3, even though they had been instructed to open them only to T2. Sensitivity analyses with a covariate defining whether links were (re)opened after T2 showed that exclusion of these participants did not alter the overall pattern of ANOVA results on primary outcome measures. Therefore, results from the full sample are presented. For all analyses, the significance level was set at α = 0.05. IBM SPSS Statistics (version 26) was used for all statistical data analyses.

## 4 Results

### 4.1 Baseline characteristics

As shown in Table 1, the *t*-/chi-square tests revealed that the demographic characteristics and baseline scores of the primary outcome measures were comparable in both groups (all *p*s > 0.05).

### 4.2 Affect- and stress-related outcome measures

A summary of the descriptive data for all affect- and stress-related outcome measures can be found in the Supplementary Table 6. The ANOVA on the effect of the interventions on momentary PA based on the PANAS-C-SF-PA revealed no significant interaction between time and group, *F*(2, 138) = 0.37, *p* = .693, partial η^*2*^ = .005. Furthermore, no significant main effect for group, *F*(1, 69) = 2.79, *p* = .100, partial η^2^ = .039, or time, *F*(2, 138) = 0.76, *p* = .470, partial η^2^ = .011, were observed. Similarly, the 3 × 2 ANOVA for momentary NA did not reveal a significant interaction between time and group, Greenhouse–Geisser *F*(1.67, 118.05) = 0.60, *p* = .525, partial η^*2*^ = .008. The main effects for group, *F*(1, 70) = 0.05, *p* = .824, partial η^*2*^ = .001, and time, Greenhouse-Geisser *F*(1.67, 118.05) = 2.75, *p* = .077, partial η^*2*^ = .038, were both non-significant. Moreover, ANOVA results indicated no significant interaction between time and group in terms of perceived stress based on the PSS-10, *F*(2, 140) = 0.05, *p* = .950, partial η^*2*^ = .001. However, the main effect for time was significant, *F*(2, 140) = 6.48, *p* = .002, partial η^*2*^ = .085. Post-hoc dependent *t*-tests revealed a decrease of PSS-10 scores from T2 to T3 across both groups, *t*(71) = 2.72, *p* = .008, as well as from T1 to T3, *t*(71) = 3.48, *p* < .001. Across groups, the difference in PSS-10 scores between T1 and T2 was non-significant, *t*(71) = 0.70, *p* = .488. There was no significant main effect for group with respect to the PSS-10, *F*(1, 70) = 0.11, *p* = .737, partial η^2^ = .002. Regarding depressive symptoms based on the BDI-II, there was no significant interaction between time and group, *F*(2, 138) = 1.76, *p* = .176, partial η^2^ = .025. There were also no significant main effects for group, *F*(1, 69) = 0.00, *p* = .964, partial η^2^ = .000, or time, *F*(2, 138) = 1.06, *p* = .348, partial η^2^ = .015.

### 4.3 Acceptance and adherence

Overall acceptance of the PPI (“What grade do you give the positive exercises?”) was rated as very good to good (*M* = 1.72, *SD* = 0.69). Table 2 presents further results on the acceptance for the PPI group. Regarding adherence, 0.0 % participants in the EG answered the item “How many positive exercises have you done?” with “None.”, 4.2 % with “Approx. 1–4.”, 33.3 % with “Approx. 5–9.”, 45.8 % with “Approx. 10–13.”, and 16.7 % with “All.”. Based on self-report, 87.5 % of participants completed the exercises entirely or mainly (see Table 2 for details). Table 5 of the Supplementary Material summarizes usage data for both groups. Usage data shows that approximately 64 % opened 10 or more of the links containing the PPI.

A summary of the results pertaining to acceptance and adherence for the active control condition can be found in the Supplementary Table 4. The main findings indicate that the overall acceptance of the factual knowledge messages was rated as slightly better than good, *M* = 1.92 (*SD* = 0.59). Furthermore, none of the CG participants answered the question “I did the factual knowledge riddles” with “not accurate”. This question was answered with “somewhat accurate” by 7.7 % of the CG participants, while 53.8 % answered “mainly accurate” and 38.5 % answered “entirely accurate”.

## 5 Discussion

The aims of this RCT were to assess the efficacy, acceptance, and adherence of a brief web-based self-help multi-component PPI in healthy adolescents. While no differential effects of the PPI on affect- and stress-related outcomes were found compared to the active control group, there was a good acceptance and adherence of the PPI. More specifically, overall acceptance was rated as positive. Regarding adherence, usage data showed that approximately 64 % of the participants opened 10 or more of the links with the PPI exercise delivered via email, which paralleled self-reported data with 63 % of participants reporting that they had done about 10 or more exercises.

The absence of differential effects of the PPI intervention on stress-and affect related outcomes in our youth sample differs from the majority of previous studies, which have demonstrated a beneficial effect of PPI on both well-being and depressive symptoms in non-clinical and clinical adult samples (Bolier et al., 2013; Chakhssi et al., 2018; Hendriks et al., 2020; Sin and Lyubomirsky, 2009) and healthy adolescent samples (Tejada-Gallardo et al., 2020). A systematic review synthesized the results of previous meta-analyses, reviews, and high-quality RCTs on PPIs with the aim to determine under which circumstances PPIs show positive effects and which methodological features influence the magnitude of the observed effects (Donaldson et al., 2021). Identified features included the quality of the study and the type of control group used. The authors found that lower-quality RCTs often overestimated the effects of PPIs (e.g., as shown in meta-analyses by Carr et al., 2021; Hendriks et al., 2020; Tejada-Gallardo et al., 2020). Moreover, studies with an active control group reported considerably smaller effect sizes than studies with no intervention in the comparison group (for meta-analyses, see Sin and Lyubomirsky, 2009; Dickens, 2017; Carrillo et al., 2019; Carr et al., 2021; Tejada-Gallardo et al., 2020). Accordingly, one plausible explanation for the absence of differential effects might be the rigorous RCT design employed in the present study, which included an active control group.

Another methodological feature of the PPI that may have contributed to the absence of differential effects is the intensity of the intervention (Donaldson et al., 2021; van Agteren et al., 2021). The overall intensity depends on factors such as the duration, delivery format, interactivity, and therapeutic guidance. In terms of duration, the time frame of 14 days can be considered as relatively short. However, while there is evidence that longer PPIs result in greater effects (e.g., Carr et al., 2021; Koydemir et al., 2021; Sin and Lyubomirsky, 2009), it is important to note that the opposite pattern has also been reported (Carrillo et al., 2019), and some studies find no effect of duration (Davis et al., 2016; Geerling et al., 2020; Hendriks et al., 2018). In addition, a systematic review on various psychological interventions including, e.g., PPI, CBT-based and mindfulness interventions, indicated that the intensity of an intervention moderates its effect size (van Agteren et al., 2021), with more intense interventions resulting in more pronounced changes. The authors argue that more intensive psychological interventions might facilitate the implementation of practices in everyday life (see also Weiss et al., 2016). In addition to the aspects mentioned, the extent to which the mode of delivery of the current PPI – namely an online self-help format – may have influenced the results of the study remains unclear in the current literature. While previous work has indicated that PPIs are feasible to deliver online (e.g., Gander et al., 2013; Mongrain and Anselmo-Matthews, 2012; Mitchell et al., 2010; Seligman et al., 2005), a meta-analysis revealed that PPIs based on conventional techniques (e.g., face-to-face) exhibited superior effectiveness compared to those utilizing technology-assisted methodologies (Koydemir et al., 2021). Donaldson et al. (2021) indicate in their review that it may prove more challenging to achieve favourable outcomes through the utilization of technology-assisted interventions in comparison with the traditional, face-to-face delivery. Besides the aspect of online implementation, another facet of the delivery mode of the intervention is how guided it is. As the PPI intervention was conducted entirely online and without guidance, this might have resulted in limited engagement and may have provided fewer opportunities for reflection or clarification than face-to-face or guided formats. This is supported by findings that guided PPIs are more effective than unguided self-help PPIs in enhancing well-being and alleviating distress in both clinical and non-clinical samples (Bolier et al., 2013; Chakhssi et al., 2018; Sin and Lyubomirsky, 2009). However, empirical studies that aim to understand the characteristics of individuals who can derive the greatest benefit from online PPIs are scarce, as are information on the specific contexts in which these interventions are most effective (e.g., Baños et al., 2017). Moreover, while, e.g., face-to-face delivery may achieve greater effects, online PPIs are easily accessible, highly scalable, and adaptable to the preferences of young target groups. Thus, if future approaches succeed in optimising the efficacy of online PPIs, even small effects could have a large impact at the population level.

In light of our findings, it should further be discussed that the majority of meta-analyses revealed a stronger effect in clinical compared to non-clinical groups (Sin and Lyubomirsky, 2009; Weiss et al., 2016; Carr et al., 2021). While participants' perceived stress level was relatively high, the mean scores obtained from measures of PA, NA, and depressive symptoms indicated low symptom burden, leaving little room for improvement. Consequently, with limited potential for improvement on most measures, the ceiling or floor effect may have contributed to the non-significance of the results in the current study (Goodwin, 2010). In this context, it could be proposed that the administration of the intervention to participants with more pronounced symptoms may result in differential effects. However, we found no differential effects on affect- and stress-related outcomes in an RCT with adolescents with a history of MD who received the same PPI as the healthy adolescents in the current study (Kaubisch et al., 2023). At this point, it should be noted that both interventions exerted a nonspecific effect on PSS-10 scores across both groups, with a decrease from T2-T3 and T1-T3. It is possible that the floor effect did not come into play here, as the baseline PSS-10 scores were higher than comparable scores in this age group in healthy adolescents (Klein et al., 2016). However, based on the current findings, it remains unclear whether the reduction in perceived stress is related to the interventions or was due to unspecific time effects. In contrast to the absent effects for the BDI-II and the PANAS-C-SF, 75 % of the participants in the EG agreed with the statement “The positive exercises have improved my mood.” mainly or entirely (vs. 55.3 % in the CG; *p* < .10) and 63.4 % agreed with the statement “The positive exercises have energised and activated me.” mainly or entirely (vs. 29 % in the CG; *p* < .05). Although only limited conclusions can be drawn from these data and explorative post-hoc analyses, it might well be the case that participants perceived an immediate positive effect of the exercises, but that these effects might not have been maintained to T2.

Furthermore, with regard to methodological aspects of the study, future studies could consider using measures that might better capture subtle changes in mental health and well-being in healthy individuals, given the possibility of floor and ceiling effects in the current study. In more detail, measures could be included that are more directly related to the content of the intervention such as measures for gratitude or positive relationships. In this context, however, the concept of well-being is not consistently defined, making it hard to assess the effect of PPIs on different aspects of well-being (Moskowitz et al., 2021).

The good adherence and acceptance outcomes in the current study align with our expectations, given the involvement of media expertise and the participatory approach during the development of the website in which the PP exercises are embedded. In terms of acceptance, it should be highlighted that the majority of participants reported that they enjoyed the PPI and would recommend it to a friend. Regarding adherence, objective usage data as well as self-report data showed that nearly two thirds of participants opened/performed 10 or more of the PP exercises delivered via email. Although no quantitative cut-offs or categorisations for adherence were predefined, in context of the digital mental health intervention literature, this can be considered a promising result regarding adherence, particularly as it is comparable to or higher than adherence rates for other online self-help interventions: Dropout and compliance rates in RCTs of internet interventions for anxiety and depression were found to range from approximately 1 % to 50 % (Christensen et al., 2009). Specifically for adolescents, completion rates in digital mental health interventions have been reported to range considerably from 10 % to 94 % (Lehtimaki et al., 2021). With an adherence rate of 64 %, our study falls within the middle range. However, it should be noted that in the current study, an unguided intervention was evaluated, and it was found that full completion rates are on average 12 % higher in guided interventions compared to unguided ones (Musiat et al., 2022).

The encouraging results in terms of acceptance and adherence underline that the PPI was delivered in a mode and with a layout that is attractive to young people. Good adherence and acceptance are important prerequisites for the efficacy of an intervention. With this in mind, the present study can inform future approaches aimed at developing or optimizing web-based PPIs.

## 6 Strengths and limitations

This study had several strengths. Firstly, its sample is very well-characterized and sufficiently large. Secondly, it employed a preregistered RCT design with an active control condition, as opposed to, for example, a waitlist-control group. Thirdly, it utilized PP exercises that have been empirically supported. Despite these and other strengths, the study is also subject to limitations, including the limited ability to ascertain the extent of the PPI's integration into the participants' lives within the context of the study. Additionally, the majority of the data was derived from self-assessments, which may be susceptible to, e.g., reporting errors or memory biases. Furthermore, although we offered a variety of PP exercises targeting different domains such as gratitude or personal strengths, individual preferences for certain types of exercises among participants were not considered as this would have reduced experimental control. Future studies should therefore examine whether consideration of the “Person-Activity-Fit” might result in beneficial outcomes (Michel et al., 2020). This model describes the manner in which the congruence between an individual's characteristics, such as preferences, and the attributes of an intervention, such as variety, influence the efficacy and subsequent well-being outcomes of a PPI.

## 7 Conclusions

While the current study did not reveal differential effects of the brief online multi-component PPI, we found positive results regarding acceptance and adherence. Further research is needed to understand the optimal methodology for the delivery of PPIs to have positive effects. In this context, it is important that future research identifies which factors related to both the characteristics of the intervention and the participants may enhance the efficacy of PPIs in healthy adolescents. Continued research into online PPIs is particularly important. Although there is evidence that online and self-help methods mitigate the impact of PPIs (Koydemir et al., 2021), this method of delivery is more feasible on a large scale (Layous et al., 2011). Future studies should examine whether a longer online self-help PPI, and providing users with the opportunity for more practice, might result in beneficial effects on the primary outcomes. As our results indicated good acceptance and adherence in our youth sample, we are encouraging further mixed methods research for evaluating the perceived usefulness and person-activity-fit, thereby providing insight into relevant moderating variables. In view of the rising prevalence of mental health problems in youth (Kieling et al., 2024), an important future goal is to provide well-accepted, effective, and highly scalable interventions such as online PPIs that also take inclusivity into account. With this in mind, the present study forms an important basis for future research aimed at the development of innovative web-based interventions that are tailored to the needs of young people.

## Ethics approval

Ethical approval for the study was granted by the Ethics Committee of the Medical Faculty at Ludwig-Maximilians-University (LMU) Munich. All procedures followed the most recent guidelines outlined in the Declaration of Helsinki. Participants received comprehensive information regarding the study's purpose and procedures before giving their written informed assent. In addition, written informed consent was obtained from the participants' legal guardians after they had been fully briefed on all elements of the study.

## Funding

This study was funded by a grant from the foundation “10.13039/501100008941Prof. Otto Beisheim Stiftung” to G.S.-K., E.G., C.P., R.P., L.F. All authors declare no conflicts of interest.

## Declaration of competing interest

All authors declare no conflicts of interest.

## Data Availability

The data collected in this study include sensitive details about participants, such as various sociodemographic characteristics. Publicly sharing the raw dataset could risk revealing individual identities, thereby violating ethical standards related to participant confidentiality. As a result, the raw data will not be publicly released. However, detailed descriptions of the methodology, materials used, and sample characteristics are provided within the article and supplementary materials (such as the Positive Psychology intervention). The intervention is also publicly accessible in German on the “ich bin alles” website. Aggregated data and additional supporting materials may be shared upon request (contact: Sara.Kaubisch@med.uni-muenchen.de).

## Appendix A Supplementary data

Supplementary material Image 1
