# Supplementary material for: A web-based approach to adolescent mental health: Randomized controlled trial of a brief Positive Psychology intervention
Source: Internet Interv. 2025 Sep 12;42:100872. doi: 10.1016/j.invent.2025.100872 (PMC12546978; doi:10.1016/j.invent.2025.100872)
Supplement: Supplementary file 1 — Supplementary material [file mmc1.docx]

**Electronic Supplementary Material**

**Power analysis**

An a priori power analysis was computed to determine the sample size required to test our hypotheses. Based on the systematic review by Donaldson et al. (2021) most of the reviews and meta-analyses report effect sizes varying between small and medium for outcomes such as well-being, depression, anxiety, stress, and quality of life for the general population and patients suffering from mental, somatic, and physical illnesses (Bolier et al., 2013; Carr et al., 2020; Chakhssi et al., 2018; Hendriks et al., 2018; Hendriks et al., 2020; Sutipan et al., 2016; Tejada-Gallardo et al., 2020; Weiss et al., 2016). Assuming a small-to-moderate effect of partial η² = .03, the power analysis indicated that *N* = 66 are required to detect an interaction effect in a repeated-measures ANOVA with a power = .80 and significance level α = .05. The current sample size can therefore be considered as sufficiently large to detect the expected effects.

**Evidence-based platform on mental health and depression in youth “ich bin alles”**

Given the current rise in mental health problems in youth, including the increased rates of manifest depression and depressive symptoms (Kieling et al., 2024; Racine et al., 2021; Thapar et al., 2022) and the urgent need of low-threshold digital information tailored to the needs of adolescents, the German information platform “ich bin alles” (English: “I am everything”; [www.ich-bin-alles.de](http://www.ich-bin-alles.de)) was launched in September 2021. This platform is operated over a website and social media platforms and was developed by the Department of Child and Adolescent Psychiatry, Psychosomatics, and Psychotherapy at the Hospital of the Ludwig-Maximilians-University (LMU) Munich, Germany, in collaboration with the Beisheim Foundation and media partners. The project aims to support healthy adolescents and adolescents with major depression, as well as their parents. “ich bin alles” provides evidence-based and self-help-orientated information on mental health and depression in adolescents. Additionally, it features hands-on support, including the 14 self-help exercises based on Positive Psychology principles, intended to enhance user engagement.

*
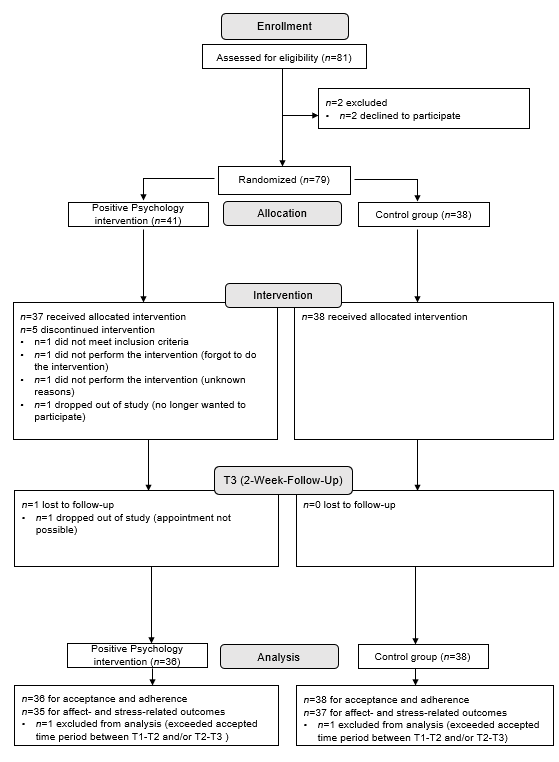
*

**Supplementary Figure 1.** *Consolidated Standards of Reporting Trials* flow diagram.

**Supplementary Table 1.** Information on the exercises of the Positive Psychology intervention in the order in which they were presented

| Domain | Title | Description |
| --- | --- | --- |
|  |  |  |
| Personal strengths | "Your strengths" | Strengths are, for example, personal qualities (such as helpfulness, humor, composure, empathy, good listening skills, sociability) or talents (such as creativity, athleticism, manual or technical skills). It is important that you know your strengths and regularly make them clear to yourself. What strengths do you have? And how do they help you when things aren't going so well? Think of specific situations in which your strengths helped you. Sometimes, it can be difficult to find your own strengths. Ask a good friend what strengths they see in you. Then write down 3 of your strengths on a piece of paper. Keep your list of strengths in your wallet, for example. And also save it in your cell phone. This way you will always be reminded of your strengths. |
| Gratitude or Engagement like flow and mindfulness | "The little things" | Even "little things" have a positive effect on our mood. Today, before you go to bed, write down three good things that happened to you throughout the day. This doesn't have to be anything "big" or "extraordinary". Try to focus on small good things in your daily life. Like, "Lunch was totally delicious," "The sun was shining," "Someone smiled at me in a friendly way." Can't think of anything about today? Then remember the past few days. |
| Gratitude or Engagement like flow and mindfulness | "Enjoy" | Take time today to consciously enjoy something. Something you normally do quickly and without thinking about it. For example, eat your dinner a little slower and savor it properly. Or consciously enjoy your shower. Afterward, think about it: What is the difference when you do something with or without enjoyment? |
| Pleasure | "Plan something beautiful" | Do one thing today that you enjoy. This can help you improve your mood and get active. For example, listen to a podcast or call a good friend. What’s important: Make this part of your daily routine. For example, set a specific reminder in your phone (e.g., "Time for a podcast today at 4:00 p.m."). |
| Pleasure | "Reward yourself" | Reward yourself regularly. You can also do something good for yourself or treat yourself just like that - even without having "achieved" anything. Think about it today: What can I do to do something good for myself? What can I treat myself to? Lie down on a meadow or treat yourself to an ice cream when the weather is good. Or let your shoulders rotate forwards and backwards for a few minutes and close your eyes. |
| Positive relationships | "Surprise others" | Do something good for one of your favorite people today. Surprise them with a spontaneous call or a nice message/GIF, for example. Small surprises can have a big impact. If you can't think of a favor or small surprise on the spur of the moment, feel free to ask them directly, "I want to do you a small favor - what would make you happy?" Often, the other person is already happy just about this question. |
| Pleasure | "Write a lucky note" | In which situation were you once really happy? Try to remember it. It can be a nice experience, a trip or a nice moment with your favorite people or your family. Recall the situation exactly. It often helps to close your eyes for a moment. Write down the situation on a piece of paper and always have your lucky note with you. Put it in your wallet, for example, so that you are always reminded of what was nice/happy. |
| Gratitude or Engagement like flow and mindfulness | "Be happy about everyday things" | There are many everyday pleasures that you often don't notice consciously. Or that you encounter more by chance. Take a look at the following list. These are all suggestions for small everyday pleasures. Again, it's important to focus on "little things." Add your own everyday pleasures to the list. (Eating a piece of cake or something else delicious, Watching the clouds, Drinking tea in the evening in a comfortable place, Giving something as a gift, + Adding another item ...) Download your list and print it out. |
| Gratitude or Engagement like flow and mindfulness | "Discover beauty" | Today, pay attention to the 3 things you particularly like in your environment or in a room. What do you find really beautiful? Record it with your (cell phone-) camera. Is it your favorite photo, a pair of shoes, the view from your room? Or something completely different? Look at the pictures you've taken again in the evening before you go to sleep. Have you ever looked so closely for something beautiful close to you? |
| Personal strengths | "More yeah" | A positive motto can also cheer you up. Take some time and read the following sentences in peace: "Don't worry, be happy!", "Life is tough but so are you!", "You are stronger than you think!", "Even what seems impossible can come true!", "Can’t do, won’t do!", "You grow with your experiences!", "Yes, I can!", "Don't dream your life, live your dreams...". Is there a suitable motto for you? Or do you already have your own motto that encourages and cheers you up? If you have found a sentence (or even several sentences), then write them down. And pin them on a place where you often pass by! |
| Gratitude or Engagement like flow and mindfulness | "Be grateful" | Today, think about what good things have happened to you in your life. What are you grateful for? These can be very different things. You can be grateful for your siblings or friends, for nice encounters with others, for the last summer vacation or for a nice teacher. What comes to your mind? Write down 3 things you are grateful for. |
| Positive relationships | "Positive vibrations" | Say something good, nice, positive to someone who means a lot to you. This will strengthen your relationship with that person and put you both in a good mood. Think of at least one good thing you can say to someone who means a lot to you. For example, to a friend, your parents, or siblings: "I like you the way you are.", "It's nice that you exist.", "I'm proud of you.", "Thank you for ... (doing this for me).", "I think it's great about you that ...", "It's lucky that you're here!", "Thank you for always supporting me.", You can also praise them for something they did well: "Bravo! You did really well." + Click here and add something yourself.  You can tell them in person or by cell phone. |
| Pleasure | "Listen yourself happy" | Music can help improve your mood. Take some time today to search your phone or the Internet for a song that will put you in a good mood. It can be a song you already know. Or maybe you'll find one you don't know yet. Ask your family or friends what songs make them feel good. When you find a song, turn it up as loud as you feel comfortable. Listen to it several times. And sing or hum along if you like. |
| Gratitude or Engagement like flow and mindfulness | "Make a fantasy journey" | While you go on the fantasy journey, you can relax and just switch off for a little moment. It lasts about 5 minutes. It is best to find a place at home where you feel comfortable for the fantasy journey. It is important that you can relax there and not be distracted by loud noises. This can be your bed or your favorite chair or sofa. Sit or lie down and relax. Feel free to close your eyes if this is comfortable for you... (fantasy journey is given) What beautiful impressions did you gather during the fantasy journey? Call them to your memory. And try to keep them for a while. |

*Note:* All interventions were delivered in German.

**Supplementary Table 2.** Information on the factual knowledge condition in the order in which it was presented

| Question | Description |
| --- | --- |
|  |  |
| "What is the shoe size of the Statue of Liberty in the USA?"^a^ | A - 518; B - 948; C - 1143  Answer: The Statue of Liberty (also known as "Lady Liberty") is one of the most famous statues in the world. Americans received it as a gift from France - a great gift (literally).  The statue stands on its own island called "Liberty Island" near New York, is 93 meters high and has 7.62 meters long feet. That would be equal to a German giant shoe size of 1143 (which of course does not exist in shoe stores). By the way, the giant feet of the statue are in giant sandals. |
| "How many hearts beat in the chest of an octopus?" ^a^ | A - 2; B - 5; C - 8  Answer: Octopuses are impressive creatures of the underwater world. They have no bones, but they do have eight arms and three hearts: one main heart for the brain and body, and two other hearts for breathing.  Octopuses move very elegantly and agilely in the water, they can fit through even the narrowest rock openings. They can also change the color and pattern of their skin, adapting perfectly to their environment - much like chameleons. |
| "Where is the highest church tower in the world?" ^a^ | A - Ulm; B - Cologne; C - Münster  Answer: The highest church tower in the world is not in Cologne, Hamburg or Berlin - but in Ulm. Ulm is a city in Baden-Württemberg - on the border with Bavaria. Ulm Cathedral, as the church is called, is 161 meters high and was completed in 1890 after a long construction period. To reach the church tower, you have to climb 768 steps. This can be quite sweaty, especially in summer. From the top, however, you have a wonderful view over Ulm's old and new town. |
| "What does Scotland have to do with the unicorn?" | Answer: Unicorns are "in". You see the mythical creatures on many products these days, for example on school backpacks or T-shirts. In Scotland, the unicorn has a very special meaning - far beyond backpacks. It is the Scottish national animal, is considered a "hero symbol" and stands for pride, freedom and purity. |
| "In which months is there a Friday the 13th?" | Answer: All months that begin with a Sunday have a Friday the 13th. As is well known, there is a superstition that this day or the number 13 brings bad luck. Some airplanes therefore do not have a 13th row of seats. The superstition associated with "Friday the 13th" goes back to different things and events. For example, Christians associate (Holy) Friday with the death of Jesus Christ. The number 13 is also considered the "devil's dozen". |
| "Why do you close your eyes when you sneeze?" | Answer: The eyes and nose are closely connected. This connection has an important protective function: closing the eyes prevents bacteria or germs from entering the eyes from the nose when sneezing. In addition, the whole body tenses automatically for a short time when sneezing, including the eye muscles. Which also causes the eyelids to close. |
| "Is a rainbow also visible at night?" | Answer: Yes, when there is a full moon and rain, it is indeed possible to see a rainbow. The moonlight is then, similar to the sunlight, divided by the raindrops into its colored individual parts. The nocturnal rainbow is however more weakly pronounced than the daylight rainbow. You have to look very closely then. |
| "Piano: Is the piano a keyboard, percussion or string instrument?" | Answer: yes, yes and yes. The piano has keys, pedals and strings. It is therefore at the same time a keyboard instrument (keys are operated to produce tones), a percussion instrument (because the strings are "struck" by a mechanism and thus made to vibrate) and a string instrument (the strings in the piano vibrate and thus produce tones). |
| "Where does the word alarm actually come from?" | Answer: The word alarm = warning sign, danger signal comes from the French "alarme" or " à l'arme!”. Translated it means: "to the weapon!", so it is originally a call, which was used by the military to wake up or for example to draw attention to an imminent danger.  Today, the alarm is still used as an emergency signal (e.g., as the wailing sound of a siren). By the way, anyone who sets off an alarm without needing to do so (e.g., for fun by alerting the fire department) is liable to prosecution. |
| "Since when do tattoos exist?" | Answer: For a very, very long time. One proof is the over 5000 year old "Iceman" named Ötzi. Ötzi wore several tattoos - but not as elaborate as today, with different colors or creative shapes. Ötzi had very "simple" line patterns on his skin. In his time, charcoal powder may have been put into puncture wounds to create color. It is also assumed that Ötzi's stitches were used to treat pain - and not as jewelry. By the way, Ötzi can be seen in a museum in Bozen (a town in South Tyrol). But be careful - the queue in front of the museum is always very long. |
| "Are there more stars in the Milky Way or more trees on Earth?" ^a^ | A - more trees; B - more stars; C - as many stars as trees.  Answer: Who would have thought: There are more trees on earth than stars in the Milky Way. Although the Milky Way contains around 100 to 300 billion stars, this is a much smaller number compared to the three trillion trees that exist on our planet. Incidentally, the tree that is most common in Germany is the spruce. A popular Christmas tree. Only the Nordmann fir is even more popular. |
| "Do mussels hurt?" | Answer: Very likely no. Researchers have found that living beings need at least a very simple brain to have feelings (whether positive or negative feelings). However, mussels do not have such a brain, but only a few nerve cells. That is why it is now believed that mussels do not feel emotions. Nevertheless, we should of course be careful with mussels, which live in waters all over the world. Because many species of mussels are unfortunately already extinct. |
| "Are humans or horses faster over a distance of 30 km?" ^a^ | A - people; B - horses; C - people and horses are equally fast at this distance.  Answer: Amazingly, horses and humans are about the same speed. At least if you follow this story: Every year, a small town in Wales hosts a race of horses and humans over about 35 km. So far there were victories of the horses as well as of the humans.  This is probably due to the fact that horses are less able to cool down at high speeds because of their weight. The "lighter" human body, on the other hand, can dissipate heat well through sweating, for example - and therefore copes better with heating, especially over longer distances. Also clear is: Over a distance of, let's say 1km, a human being has no chance against a horse. |
| "Does a woodpecker have a headache when it knocks?" | Answer: The woodpecker knocks and hacks a cave in the tree to nest there later. A strenuous job. But is it so strenuous that it gives him a headache? No, researchers have discovered. There are processes that protect the woodpecker's brain when it knocks: For example, the woodpecker's skull bone contains soft tissues that absorb the impact or rebound of tapping. And also the beak, or more precisely the different lengths of the beak, buffers the up and down impact when tapping. |

*Note:* All factual knowledge messages were delivered in German.

1. This factual knowledge message contained a choice of possible correct answers.


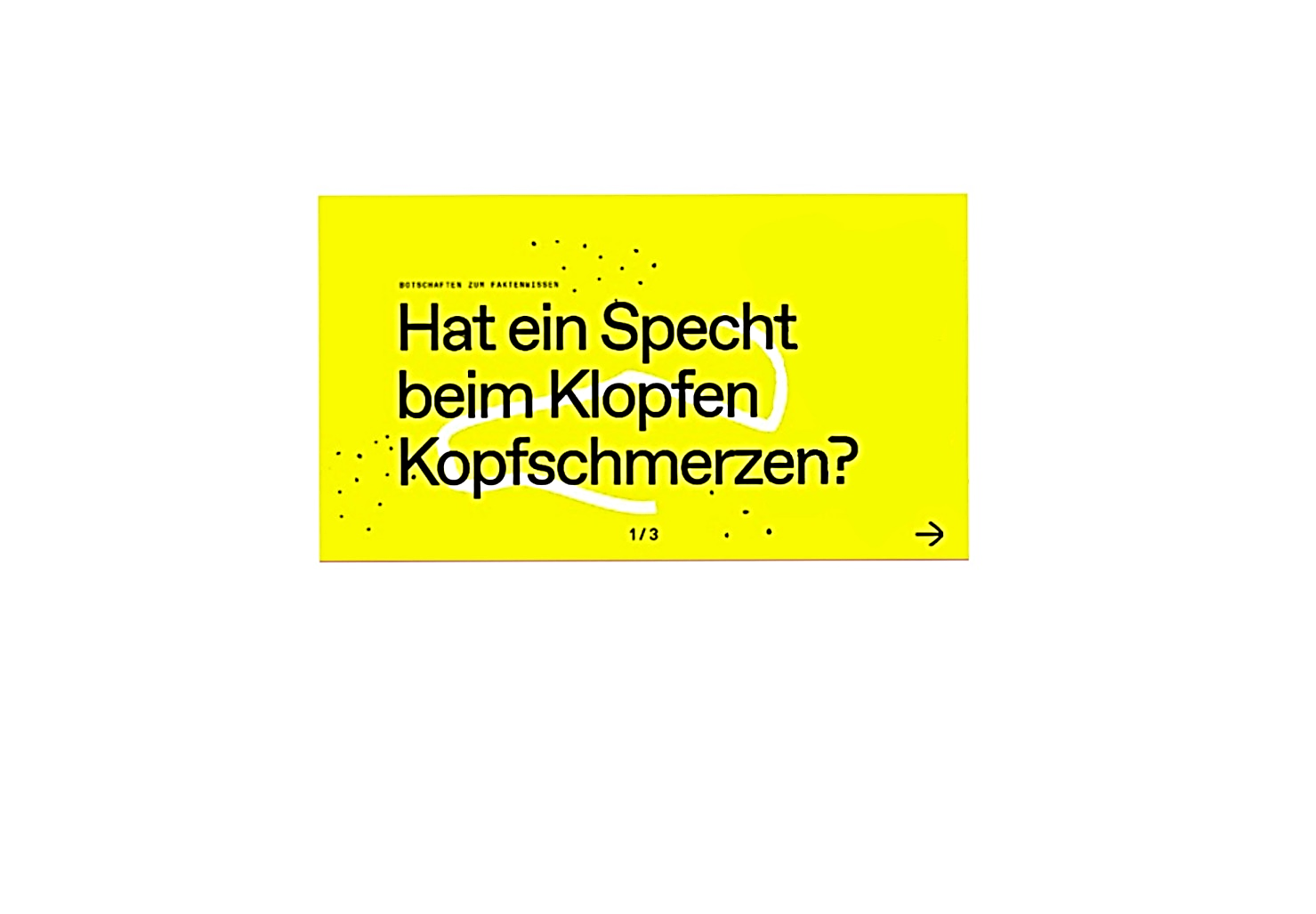


**
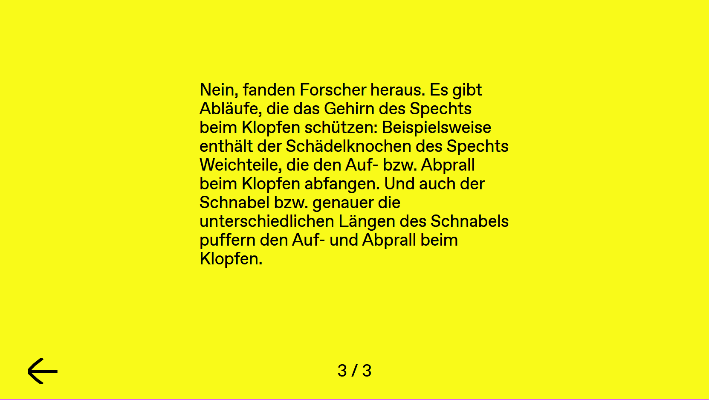

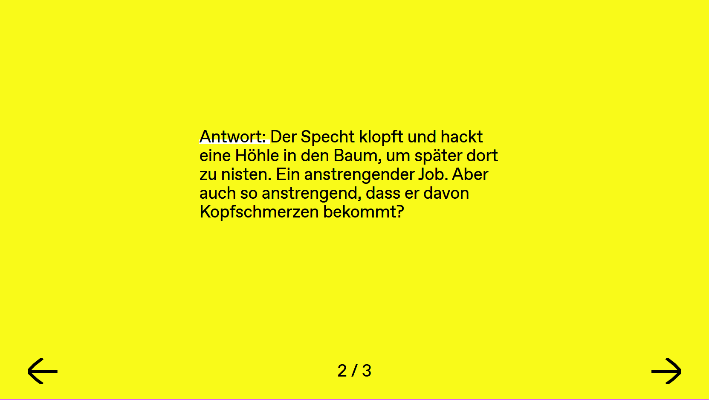
**

**Supplementary Figure 2.** Example of a factual knowledge message (a graphic of a woodpecker had to be removed due to issues related to image rights for publication).

**Supplementary Table 3.** Psychometric properties of the measures applied

| Measure | Instrument | Reliability/validity |
| --- | --- | --- |
| Depressive symptoms | BDI-II | Based on a meta-analysis (Stockings et al., 2015) the pooled estimate for internal reliability is .86 and the discriminative validity pooled estimate for sensitivity and specificity is .81. |
| Diagnostic interview | Kinder-DIPS | The interview has good retest and interrater reliabilities (Cohens Kappa ≥ 0.90) and good validity (Adornetto et al., 2008; Neuschwander et al., 2013). |
| IQ test | CFT-20-R | The intelligence test has excellent reliability (Cronbach’s α = .96; Weiß, 2006). |
| Positive and negative affect | PANAS-C-SF | Cronbach’s alpha for the reduced 5-item Positive Affect scale is .86 and for the shortened 5-item Negative Affect scale is .82 (Ebesutani et al., 2012). |
| Stress in everyday life | PSS-10 | Internal consistencies in a nonclinical sample = .88 as well as a clinical sample = .89 were good to very good (Schneider et al., 2020). |

*Note:* BDI-II = Beck Depression Inventory-II. Kinder-DIPS = Diagnostisches Interview bei psychischen Störungen im Kindes- und Jugendalter [diagnostic interview for mental disorders in childhood and adolescence]. CFT-20-R = Culture Fair Intelligence Test-Revised. PANAS-C-SF = Positive and Negative Affect Schedule for Children-Short Form. PSS-10 = Perceived Stress Scale.

**Supplementary Table 4.** Results for items of the evaluation questionnaire for the factual knowledge messages (in rounded %)

| **Items** | Mean (SD) | entirely accurate | mainly accurate | somewhat accurate | not accurate |
| --- | --- | --- | --- | --- | --- |
| **Acceptance** |  |  |  |  |  |
| What grade do you give the factual knowledge messages? | 1.92 (0.59) | n.a. | n.a. | n.a. | n.a. |
| It was fun to read the factual knowledge messages. | n.a. | **68.4** | 21.1 | 7.9 | 2.6 |
| I would recommend the factual knowledge messages to other youth. | n.a. | **42.1** | 36.8 | 15.8 | 5.3 |
| I was able to apply the factual knowledge well in my everyday life. | n.a. | 13.2 | 15.8 | 42.1 | **28.9** |
| The factual knowledge messages have improved my mood. | n.a. | 13.2 | **42.1** | 28.9 | 15.8 |
| The factual knowledge messages have energised and activated me. | n.a. | 21.1 | 7.9 | **55.3** | 15.8 |
| **Adherence** |  |  |  |  |  |
| I did the factual knowledge riddles.^a^ | n.a. | 38.5 | **53.8** | 7.7 | 0 |

*Note:* Using a four-point rating scale (0: not accurate, 1: somewhat accurate, 2: mainly accurate, 3: entirely accurate); %; except for grade (grade 1: very good, grade 6: insufficient based on the German school grading system); *n* = 38 (except for the item “I did the factual knowledge riddles.” *n* = 26). Most frequent answers are shown in bold.

1. Some factual knowledge messages also contained interactive tasks with a choice of possible correct answers.

**Supplementary Table 5.** Information on usage data for both groups

| Answer categories for the question "How many positive exercises have you done?" | Number of interventions opened, *n* (%) | Experimental group: Positive Psychology intervention  (*n* = 36) | Control group  (*n* = 38) |
| --- | --- | --- | --- |
| "Approx. 1-4 positive exercises." |  |  |  |
|  | 1 | 2 (5.6%) | 0 (0.0%) |
|  | 2 | 0 (0.0%) | 1 (2.6%) |
|  | 3 | 3 (8.3%) | 2 (5.3%) |
|  | 4 | 1 (2.8%) | 2 (5.3%) |
| "Approx. 5-9 positive exercises." |  |  |  |
|  | 5 | 1 (2.8%) | 0 (0.0%) |
|  | 6 | 0 (0.0%) | 0 (0.0%) |
|  | 7 | 2 (5.6%) | 0 (0.0%) |
|  | 8 | 1 (2.8%) | 1 (2.6%) |
|  | 9 | 3 (8.3%) | 0 (0.0%) |
| "Approx. 10-13 positive exercises." |  |  |  |
|  | 10 | 0 (0.0%) | 1 (2.6%) |
|  | 11 | 1 (2.8%) | 5 (13.2%) |
|  | 12 | 1 (2.8%) | 4 (10.5%) |
|  | 13 | 3 (8.3%) | 3 (7.9%) |
| "All." |  |  |  |
|  | 14 | 18 (50.0%) | 19 (50.0%) |

*Note:* The answer categories of the self-report inventory designed by the investigators are attached here for better comparison between self-report and usage data.

**Supplementary Table 6.** Means and standard deviations for primary outcomes

| Measure | T1  *M* (*SD*) | T2  *M* (*SD*) | T3  *M* (*SD*) |
| --- | --- | --- | --- |
| **Experimental Group: Positive Psychology Intervention** |  |  |  |
| PANAS-C-SF: Positive Affect | 16.14 (3.10) | 16.22 (3.60) | 16.50 (4.31) |
| PANAS-C-SF: Negative Affect | 5.47 (1.46) | 5.38 (1.57) | 5.64 (1.15) |
| PSS-10 | 21.51 (6.93) | 21.28 (6.35) | 19.79 (5.50) |
| BDI-II | 3.83 (6.61) | 3.51 (4.46) | 3.03 (2.96) |
| **Control Group** |  |  |  |
| PANAS-C-SF: Positive Affect | 16.97 (3.85) | 17.66 (4.04) | 17.38 (3.44) |
| PANAS-C-SF: Negative Affect | 5.44 (1.31) | 5.29 (0.77) | 5.82 (1.39) |
| PSS-10 | 22.61 (5.21) | 21.97 (5.34) | 20.55 (4.83) |
| BDI-II | 4.26 (4.71) | 3.26 (3.59) | 3.13 (4.18) |

*Abbreviations:* PANAS-C-SF = Positive and Negative Affect Schedule for Children-Short Form, PSS-10 = Perceived Stress Scale, BDI-II = Beck Depression Inventory - Second Edition.

*Note:* In all measures, higher values represent a higher expression of the assessed construct.

**Supplementary References**

Adornetto, C., In-Albon, T. & Schneider, S. (2008). Diagnostik im Kindes- und Jugendalter anhand strukturierter Interviews: Anwendung und Durchführung des Kinder-DIPS. *Klinische Diagnostik und Evaluation*, *1*(4), 363-377.

Bolier, L., Haverman, M., Westerhof, G. J., Riper, H., Smit, F., & Bohlmeijer, E. (2013). Positive psychology interventions: a meta-analysis of randomized controlled studies. *BMC public health*, *13*, 119. <https://doi.org/10.1186/1471-2458-13-119>

Carr, A., Cullen, K., Keeney, C., Canning, C., Mooney, O., Chinseallaigh, E., & O’Dowd, A. (2021). Effectiveness of positive psychology interventions: a systematic review and meta-analysis. *The journal of positive psychology*, *16*(6), 749-769. <https://doi.org/10.1080/17439760.2020.1818807>

Chakhssi, F., Kraiss, J. T., Sommers-Spijkerman, M., & Bohlmeijer, E. T. (2018). The effect of positive psychology interventions on well-being and distress in clinical samples with psychiatric or somatic disorders: a systematic review and meta-analysis. *BMC psychiatry*, *18*(1), 211. <https://doi.org/10.1186/s12888-018-1739-2>

Donaldson, S. I., Cabrera, V., & Gaffaney, J. (2021). Following the science to generate well-being: Using the highest-quality experimental evidence to design interventions. *Frontiers in Psychology*, *12*, 739352. <https://doi.org/10.3389/fpsyg.2021.739352>

Ebesutani, C., Regan, J., Smith, A., Reise, S., Higa-McMillan, C., & Chorpita, B. F. (2012). The 10-item positive and negative affect schedule for children, child and parent shortened versions: application of item response theory for more efficient assessment. *Journal of Psychopathology and behavioral Assessment*, *34*(2), 191-203.

Hendriks, T., Schotanus-Dijkstra, M., Hassankhan, A., Graafsma, T. G. T., Bohlmeijer, E., & de Jong, J. (2018). The efficacy of positive psychological interventions from non-western countries: a systematic review and meta-analysis. *International Journal of Wellbeing*, *8*(1). <https://doi.org/10.5502/ijw.v8i1.711>

Hendriks, T., Schotanus-Dijkstra, M., Hassankhan, A., de Jong, J., & Bohlmeijer, E. (2020). The efficacy of multi-component positive psychology interventions: A systematic review and meta-analysis of randomized controlled trials. *Journal of Happiness Studies: An Interdisciplinary Forum on Subjective Well-Being, 21*(1), 357–390. <https://doi.org/10.1007/s10902-019-00082-1>

Kieling, C., Baker-Henningham, H., Belfer, M., Conti, G., Ertem, I., Omigbodun, O., Rohde, L. A., Srinath, S., Ulkuer, N., & Rahman, A. (2011). Child and adolescent mental health worldwide: evidence for action. *Lancet (London, England)*, *378*(9801), 1515–1525. <https://doi.org/10.1016/S0140-6736(11)60827-1>

Neuschwander, M., In-Albon, T., Adornetto, C., Roth, B., & Schneider, S. (2013). Interrater-Reliabilität des Diagnostischen Interviews bei psychischen Störungen im Kindes- und Jugendalter (Kinder-DIPS) [Interrater reliability of the «Diagnostic Interview bei psychischen Störungen im Kindes- und Jugendalter (Kinder-DIPS)]. *Zeitschrift fur Kinder- und Jugendpsychiatrie und Psychotherapie*, *41*(5), 319–334. <https://doi.org/10.1024/1422-4917//a000247>

Racine, N., McArthur, B. A., Cooke, J. E., Eirich, R., Zhu, J., & Madigan, S. (2021). Global Prevalence of Depressive and Anxiety Symptoms in Children and Adolescents During COVID-19: A Meta-analysis. *JAMA pediatrics*, *175*(11), 1142–1150. <https://doi.org/10.1001/jamapediatrics.2021.2482>

Schneider, E. E., Schönfelder, S., Domke-Wolf, M., & Wessa, M. (2020). Measuring stress in clinical and nonclinical subjects using a German adaptation of the Perceived Stress Scale. *International journal of clinical and health psychology : IJCHP*, *20*(2), 173–181. <https://doi.org/10.1016/j.ijchp.2020.03.004>

Stockings, E., Degenhardt, L., Lee, Y. Y., Mihalopoulos, C., Liu, A., Hobbs, M., & Patton, G. (2015). Symptom screening scales for detecting major depressive disorder in children and adolescents: a systematic review and meta-analysis of reliability, validity and diagnostic utility. *Journal of affective disorders*, *174*, 447–463. <https://doi.org/10.1016/j.jad.2014.11.061>

Sutipan, P., Intarakamhang, U., & Macaskill, A. (2017). The impact of positive psychological interventions on well-being in healthy elderly people. *Journal of Happiness Studies: An Interdisciplinary Forum on Subjective Well-Being, 18*(1), 269–291. [https://doi.org/10.1007/s10902-015-9711-z](https://psycnet.apa.org/doi/10.1007/s10902-015-9711-z)

Tejada-Gallardo, C., Blasco-Belled, A., Torrelles-Nadal, C., & Alsinet, C. (2020). Effects of School-based Multicomponent Positive Psychology Interventions on Well-being and Distress in Adolescents: A Systematic Review and Meta-analysis. *Journal of youth and adolescence*, *49*(10), 1943–1960. <https://doi.org/10.1007/s10964-020-01289-9>

Thapar, A., Eyre, O., Patel, V., & Brent, D. (2022). Depression in young people. *Lancet (London, England)*, *400*(10352), 617–631. <https://doi.org/10.1016/S0140-6736(22)01012-1>

Weiss, L. A., Westerhof, G. J., & Bohlmeijer, E. T. (2016). Can We Increase Psychological Well-Being? The Effects of Interventions on Psychological Well-Being: A Meta-Analysis of Randomized Controlled Trials. *PloS one*, *11*(6), e0158092. <https://doi.org/10.1371/journal.pone.0158092>

Weiß, R. H. (2006). *CFT 20-R: Grundintelligenztest Skala 2-Revision*. Hogrefe.
